# Association between radiotherapy and risk of second primary malignancies in patients with resectable lung cancer: a population-based study

**DOI:** 10.1186/s12967-022-03857-y

**Published:** 2023-01-09

**Authors:** Bolun Zhou, Ruochuan Zang, Peng Song, Moyan Zhang, Fenglong Bie, Guangyu Bai, Yuan Li, Qilin Huai, Yuning Han, Shugeng Gao

**Affiliations:** 1 Department of Thoracic Surgery, National Cancer Center/National Clinical Research Center for Cancer/Cancer Hospital, Chinese Academy of Medical Sciences and Peking Union Medical College, Beijing, China; 2 Department of General Thoracic Surgery, General Hospital of Ningxia Medical University, Ningxia, China

**Keywords:** Radiotherapy, Lung cancer, Second primary malignancies, Population study

## Abstract

**Background:**

The most common form of treatment for non-metastatic lung cancer is surgery-based combination therapy, which may also include adjuvant radiotherapy or chemotherapy. Second primary malignancies (SPMs) are uncommon but significant radiation side effects in patients with resectable lung cancer, and SPMs have not been adequately investigated. Our study aims to assess the correlations of radiotherapy with the development of SPMs in patients with resectable lung cancer.

**Methods:**

We screened for any primary malignancy that occurred more than five years after the diagnosis of resectable lung cancer. Based on the large cohort of the Surveillance, Epidemiology and End Results database, radiotherapy-correlated risks were estimated using the Poisson regression analysis and the cumulative incidence of SPMs was calculated using Fine-Gray competing risk regression analysis.

**Results:**

Among the 62,435 patients with non-metastatic lung cancer undergoing surgery, a total of 11,341 (18.16%) patients have received radiotherapy. Our findings indicated that radiotherapy was substantially related to a high risk of main second solid malignancies (RR = 1.21; 95%CI, 1.08 to 1.35) and a negligible risk of main second hematologic malignancies (RR = 1.08; 95%CI, 0.84 to 1.37). With the greatest number of patients, the risk of acquiring a second primary gastrointestinal cancer was the highest overall (RR = 1.77; 95 percent CI, 1.44 to 2.15). The cumulative incidence and standardized incidence ratios of SPMs revealed similar findings. Furthermore, the young and the elderly may be more vulnerable, and the highest risk of acquiring most SPMs was seen more than ten years after lung cancer diagnosis. Additionally, more attention should be paid to the second primary gastrointestinal cancer in young individuals with resectable lung cancer.

**Conclusion:**

After receiving radiotherapy, an increased risk of developing second primary solid and gastrointestinal cancers was observed for patients with resectable lung cancer. The prevention of SPMs associated with radiotherapy requires further attention.

**Supplementary Information:**

The online version contains supplementary material available at 10.1186/s12967-022-03857-y.

## Background

Lung cancer is one of the most prevalent cancers and the main cause of cancer-specific deaths globally [1, 2]. In 2020, it is anticipated that lung cancer accounts for approximately 1.8 million deaths and 2.2 million new cases worldwide, with the United States accounting for more than ten percent of deaths and new cases [3]. Multiple treatment modalities are utilized in the therapy of lung cancer, including surgical excision, radiotherapy, chemotherapy, immunotherapy and targeted therapy [4–6]. Radiotherapy is considered the only treatment strategy that can be used in all stages of lung cancer [7]. In general, to achieve better therapeutic outcomes, radiotherapy is used as the adjuvant therapy after surgery for lung cancer [8]. For instance, Yun et al. found that adjuvant radiotherapy might have had an additional effect on pN2 non-small cell lung cancer (NSCLC) with multiple N2 metastasis or extranodal invasion, resulting in a survival benefit [9]. Compared with adjuvant radiotherapy, preoperative radiotherapy is less common for lung cancer, and its main purpose is to improve prognosis by reducing the risk of local tumor recurrences, which may be particularly helpful in advanced lung cancer [10, 11]. A population-based study found that appropriately using radiotherapy may contribute to an 8.3% improvement in 5-year local control and a 4% increase in survival [12]. However, radiotherapy may also result in a number of adverse effects that could influence how well patients with lung cancer respond to treatment.

Second primary malignancies (SPMs) related to radiotherapy are rare but significant long-term complications that need to be thoroughly evaluated prior to radiation. Emerging studies have demonstrated that radiotherapy may substantially increase the risk of developing SPM in multiple cancers, such as breast cancer [13], nasopharyngeal carcinoma [14] and prostate cancer [15]. And some studies have investigated the optimal doses and theoretical strategies to reduce the risk of SPM for patients receiving radiotherapy [16–18]. The annual incidence of developing second primary lung cancer (SPLC) was 1.10% per patient, with an exceptionally high risk among young women with lung cancer [19]. For the treatment of lung cancer, some investigations have revealed that radiotherapy elevated the risk of developing SPMs, whereas others have shown contradictory results. For example, among NSCLC patients, a recent cohort study demonstrated that irradiation increased the risk of developing SPM by 6% [20]. For patients with fully resected NSCLC, another study did not advise routine postoperative radiation [21]. On the other hand, two cohort studies reported that radiotherapy may result in a lower risk of developing SPM, including prostate cancer and thyroid cancer, among lung cancer patients [22]. However, it has not been sufficiently addressed whether developing SPMs is a severe adverse event following radiotherapy for lung cancer, based on the sparse and contradictory findings of earlier research.

In our research, using data from Surveillance, Epidemiology and End Results (SEER) database, we intended to use different approaches to evaluate the radiotherapy-correlated risk of developing individual SPMs for lung cancer, which was rarely studied and had significant implications for radiotherapy in lung cancer treatment.

## Methods

### Participant cohorts

Using the data from nine registries of SEER database, participants diagnosed with lung cancer as the first primary cancer were enrolled from 1975 to 2018. The diagnoses of patients enrolled were pathologically confirmed and defined using the ICD-O-3 site codes (C34.0 to C34.9). The detailed codes for all cancer in this analysis were presented in Table S1. Patients with tumors of localized and regional stages at diagnosis were enrolled. The following were the exclusion criteria: patients under the age of twenty, patients with distant metastasis, patients not receiving the surgery, patients receiving radiotherapy other than external-beam radiotherapy, and those for whom race, tumor stage, age, surgery, radiotherapy, survival time or survival status information were unavailable. Patients with survival time less than five and two years after the diagnosis of the first primary cancer were also excluded in the second solid and hematologic malignancy cohorts, respectively. The survival time cut-off was based on the minimal latency period for radiation-induced tumorigenesis [23].

The use of anonymized, publicly accessible data did not need ethical approval. Access to and use of data from the SEER database did not need patient consent. This study followed the Strengthening the Reporting of Observational Studies in Epidemiology (STROBE) reporting guideline for cohort studies.

### Outcome and follow-up

The primary outcome of this study was defined as the development of SPM (second solid malignancies or hematologic malignancies) five or two years after the diagnosis of the first primary malignancy. We excluded second solid malignancies with fewer than 50 cases in the cohort with at least 5 years of follow-up, and second hematologic malignancies with fewer than 10 cases in a second cohort with at least 2 years of follow-up. In the subsequent analysis, we first evaluated the risk for all the second solid malignancies or hematologic malignancies and separately estimated the risk for each SPM. The SPMs were categorized using the ICD-O-3 site codes and detailed codes were presented in Table S1. Follow-up for second solid and hematologic malignancies began 5 and 2 years after diagnosis of the first primary cancer, respectively. And follow-up ended at all-cause death, the last follow-up, after a 30-year follow-up, SPM diagnosis, or December 31, 2018, whichever occurred first.

### Statistical analysis

Poisson regression analysis was conducted to estimate the relative risks (RRs) and 95% CIs of developing SPMs for lung cancer patients according to the administration of radiotherapy (RT vs NRT), and RRs were adjusted for gender and age at diagnosis of the first primary cancer. Next, using the Poisson regression analysis with SEER*Stat (version 8.4.0.1), the standardized incidence ratios (SIRs) and 95% confidence intervals (CIs) were then computed. The SIR was the ratio of the incidence of SPMs among patients with lung cancer to the incidence of corresponding primary malignancies in the general population of the United States. In the subgroup analysis, we stratified patients according to their clinical characteristics and calculated the RRs and SIRs of each subgroup.

With the use of Fine-Gray competing risk regression analysis, we assessed the cumulative incidence of developing SPMs. The competing events for SPM occurrence were all-cause death and non-SPM occurrence. In addition, the corresponding risk model was built to calculate the hazard ratios (HRs) and 95% CIs, which were adjusted for gender and age at diagnosis of the first primary cancer. In this study, the statistically significant threshold was P < 0.05 and all of the statistical analyses were performed with SEER*Stat (version 8.4.0.1) and R software (version 4.0.4).

## Results

### Patient characteristics

**Fig. 1:**
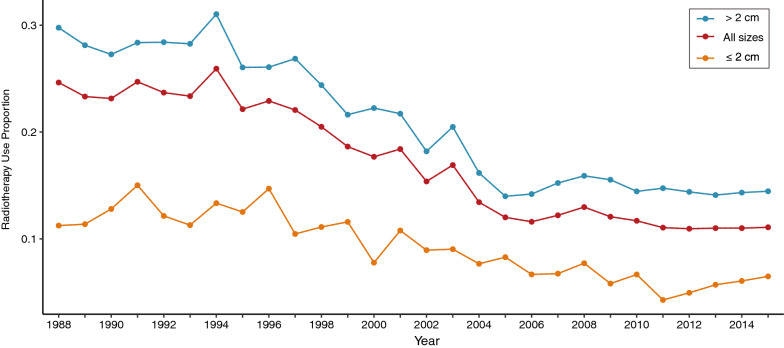
The trends in the proportion of radiotherapy application for bronchus and lung cancer in the SEER 9 (1975–2018) database, stratified by tumor size

From 1975 to 2018, we have identified 62,435 patients with nonmetastatic lung cancer in this study. Of these, a total of 11,341 (18.16%) patients have received radiotherapy, with larger tumor size, higher tumor grade, and shorter follow-up time than the no-radiotherapy subgroup (Table S2). Overall, the proportion of using radiotherapy reached its maximum rate in 1994 (25.9%) and then declined to 11.1% in 2015. And radiotherapy use proportion of patients with larger tumor sizes was higher than those with smaller sizes (Fig. 1). Next, for the analysis of second solid and hematologic malignancies, we identified 30,290 patients who survived for at least 5 years  and 46,202 patients who survived for at least 2 years in two final cohorts, respectively. As for the radiotherapy subgroups in these two final cohorts, the median follow-up time was 118 and 66 months, respectively. The patient characteristics were shown in Table 1. After a latency of 5 years, a total of 264 patients (7.4%) in the radiotherapy group and 1551 patients (5.8%) in the no-radiotherapy subgroup developed main second solid malignancies. After a latency of 2 years, a total of 56 patients (0.9%) in the radiotherapy group and 314 patients (0.8%) in the no-radiotherapy subgroup developed main second hematologic malignancies. The detailed characteristics of patients who developed main second solid and hematologic malignancies were shown in Table S3.

### Radiotherapy-correlated risks of second primary malignancies

**Fig. 2:**
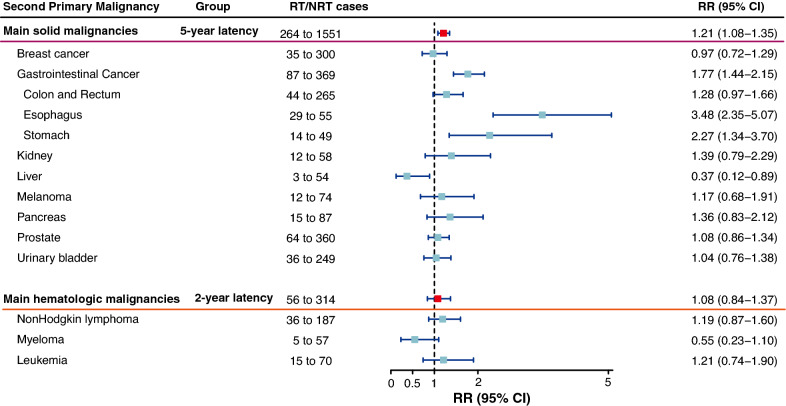
The relative risk and 95% confidence interval for main second primary malignancies, adjusted for gender and age. The analysis of prostate cancer was only for men. *RT* radiotherapy, *NRT* no radiotherapy, *RR* relative risk, *CI* confidence interval

In the two cohorts with different latency periods, radiotherapy was substantially related to a high risk of main second solid malignancies (RR = 1.21; 95%CI, 1.08 to 1.35) and a negligible risk of main second hematologic malignancies (RR = 1.08; 95%CI, 0.84 to 1.37; Fig. 2). In organ-specific analyses, radiotherapy-correlated risk was especially elevated for cancers of the esophagus (RR = 3.48; 95%CI, 2.35 to 5.07), stomach (RR = 2.27; 95%CI, 1.34 to 3.70) and colon and rectum (RR = 1.28; 95%CI, 0.97 to 1.66; Fig. 2). However, for cancers of breast (RR = 0.97; 95%CI, 0.72 to 1.29) and urinary bladder (RR = 1.04; 95%CI, 0.76 to 1.38), the radiotherapy-correlated risk was nonsignificant. In different hematologic malignancies, radiotherapy was insignificantly associated with the risk of any type of second hematologic malignancies, such as non-Hodgkin lymphoma (RR = 1.19; 95%CI, 0.87 to 1.37; Fig. 2).

Similar results were observed in multivariable competing risk regression analysis. Radiotherapy was also related to an elevated risk of developing main solid malignancies (adjusted HR, 1.16; 95%CI, 1.04 to 1.29; P = 0.03) but not related to the risk of main hematologic malignancies (adjusted HR, 1.04; 95%CI, 0.82 to 1.32; P = 0.78; Table S4). Among each type of solid cancer, the radiotherapy-correlated risk was greatly increased for cancers of the esophagus (adjusted HR, 3.31; 95%CI, 2.25 to 4.87; P < 0.01) and stomach (adjusted HR, 2.18; 95%CI, 1.32 to 3.59; P = 0.01), but the risk was nonsignificant for cancers of colon and rectum (adjusted HR, 1.22; 95%CI, 0.93 to 1.59; P = 0.24; Table S4).

### Cumulative incidences of second primary malignancies

**Fig. 3:**
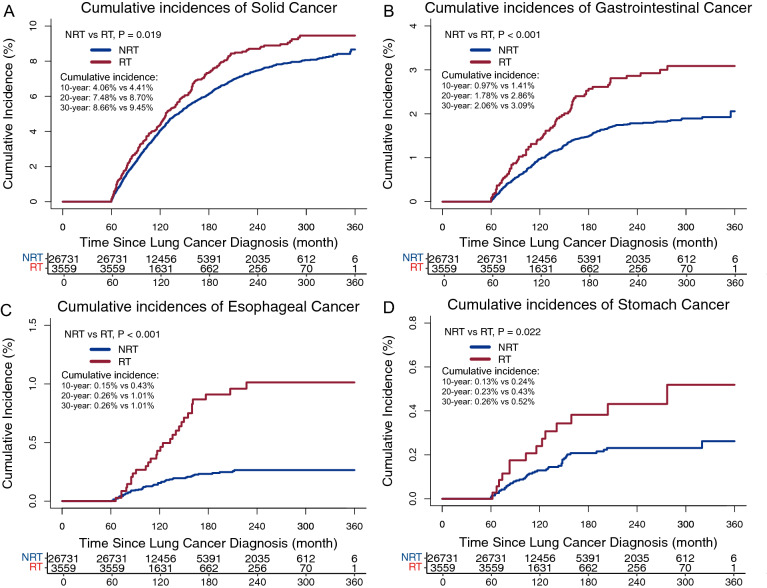
The comparisons of cumulative incidence of second primary solid cancer (**A**), gastrointestinal cancer (**B**), esophageal cancer (**C**) and stomach cancer (**D**) between subgroups stratified by radiotherapy use. The Fine-Gray test determined the P values. RT, radiotherapy; NRT, no radiotherapy

Thirty years following the diagnosis of bronchus and lung cancer, the cumulative incidences of main solid malignancies were 9.45% for patients receiving radiotherapy and 8.66% for those without radiotherapy (P = 0.019, Fig. 3A). Specifically, the 30-year cumulative incidences of radiotherapy subgroup and no-radiotherapy subgroup were significantly different for gastrointestinal cancer (3.09% vs 2.06%; P < 0.001; Fig. 3B), esophageal cancer (1.01% vs 0.26%; P < 0.001; Fig. 3C), and stomach cancer (0.52% vs 0.26%; P = 0.022; Fig. 3D). However, no difference in the 30-year cumulative incidences was observed between the two subgroups for colorectal cancer (1.56% vs 1.53%; P = 0.429, Additional file 1: Figure S1).

### Radiotherapy-related risks in different subgroups

**Fig. 4:**
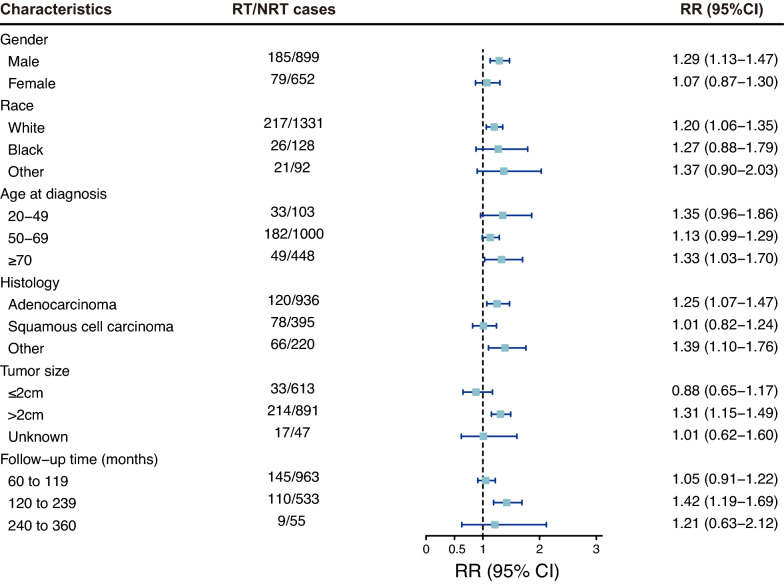
The relative risk and 95% confidence interval for main second primary cancer among subgroups stratified by different characteristics. *RT* radiotherapy, *NRT* no radiotherapy, *RR* relative risk, *CI* confidence interval

To analyze the risk of SPMs owing to radiotherapy, patients with lung cancer were subsequently stratified into different subgroups based on different characteristics. In most subgroups, the administration of radiotherapy increased the risk of developing SPMs. For main solid malignancies, the radiotherapy-correlated risk was higher in males (RR = 1.29; 95%CI, 1.13 to 1.47) than in females (RR = 1.07; 95%CI, 0.87 to 1.30; Fig. 4). A higher risk of developing main solid cancers was also observed in patients aged 20 to 49 (RR = 1.35; 95%CI, 0.96 to 1.86) and more than 70 (RR = 1.33; 95%CI, 1.03 to 1.70; Fig. 4). As for patients with different latency periods, the radiotherapy-correlated risk was the highest in the middle latency period (120 to 239 months; RR = 1.42; 95%CI, 1.19 to 1.69) and showed a declining trend in the late latency (240 to 360 months; RR = 1.21; 95%CI, 0.63 to 2.12; Fig. 4).

**Fig. 5:**
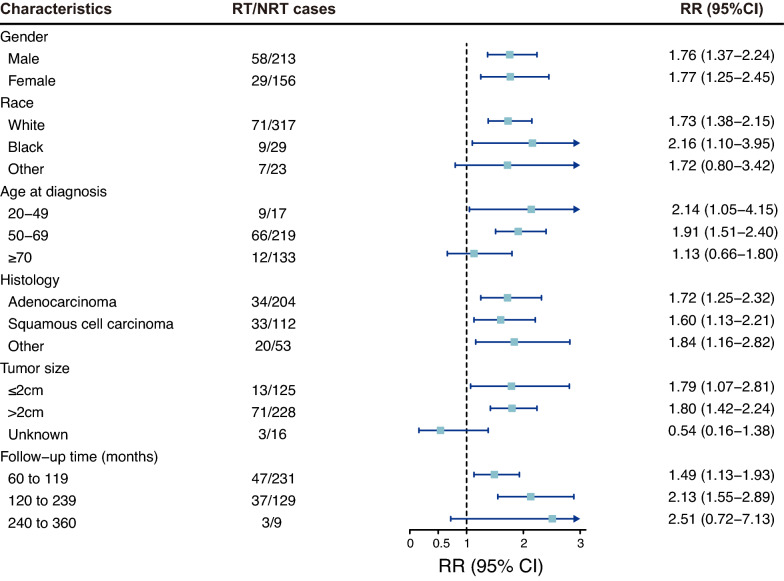
The relative risk and 95% confidence interval for second primary gastrointestinal cancer among subgroups stratified by different characteristics. *RT* radiotherapy, *NRT* no radiotherapy, *RR* relative risk, *CI* confidence interval

We then primarily focused on three different gastrointestinal malignancies in the subgroup analyses in light of the results of radiotherapy-correlated risks and cumulative incidences indicated above. Among these three types of cancers and the combined gastrointestinal cancer, females (RR = 6.04; 95%CI, 2.87 to 12.33) had a much higher risk of developing esophageal cancer than males (RR = 2.85; 95%CI, 1.79 to 4.44; Additional file 1: Figure S2), but the risk was barely different between males and females in other gastrointestinal cancers. The radiotherapy-correlated risks for developing gastrointestinal cancers peaked in patients aged 20 to 49 (RR = 2.14; 95%CI, 1.05 to 4.15) and presented a downward trend with increasing age (50–69 years, RR = 1.91; 95%CI, 1.51 to 2.40; > 70 years, RR = 1.13; 95%CI, 0.66 to 1.80; Fig. 5). Specifically, similar patterns were observed in cancer of the stomach (Additional file 1: Figure S3) and colon and rectum (Additional file 1: Figure S4), but patients aged 50 to 69 had the highest risk of developing esophageal cancer (RR = 3.84; 95%CI, 2.49 to 5.84; Additional file 1: Figure S2). As for the latency periods, the increasing risks of developing gastrointestinal cancers were accompanied by an increase in the latency time. Furthermore, the lowest risk of developing gastrointestinal cancer was observed in the subgroup with the shortest latency time (60 to 119 months), which was the same as the risk of developing main solid malignancies (Figs. 4, 5). In addition, the radiotherapy-correlated risk was similar among patients stratified by other characteristics in gastrointestinal cancers, such as race and histology.

### Standardized incidence ratios of second primary malignancies

Compared to patients who received no radiotherapy, SIRs increased more for main solid malignancies (1.30; 95%CI, 1.15 to 1.47) and main hematologic malignancies (1.41; 95%CI, 1.10 to 1.79) among patients receiving radiotherapy (Table S5). For gastrointestinal cancers, SIRs were higher in the radiotherapy subgroup (2.08; 95%CI, 1.67 to 2.55) than in the no-radiotherapy subgroup (1.16; 95%CI, 1.05 to 1.28). Specifically, compared to patients who received no radiotherapy, the SIR for esophageal cancer was much higher among patients treated with radiotherapy (8.14; 95%CI, 5.57 to 11.49). And the SIR of developing stomach cancer increased more in the radiotherapy subgroup (2.12; 95%CI, 1.09 to 3.70) than in the other one (1.13; 95%CI, 0.84 to 1.48). The findings of SIRs were similar to RRs and cumulative incidences in our study.

## Discussion

In the treatment of lung cancer patients undergoing surgery, as a crucial therapeutic approach, radiotherapy has a well-established place. Although technological advancements have minimized the unintentional irradiation of normal tissues surrounding the tumor, several studies have revealed an elevated risk of SPMs following radiotherapy for some types of cancer. In the present research, using a variety of approaches to analyze data from a large population cohort, we are the first to unveil the comprehensive correlation between radiotherapy and the risk of developing individual SPMs in resectable lung cancer patients. We learned the following significant and fascinating information: First, radiotherapy may significantly raise the chance of second primary solid tumors, particularly gastrointestinal malignancies, among patients with lung cancer who have already undergone surgery. Second, a higher cumulative risk of radiotherapy was particularly observed for developing gastrointestinal cancers, which was similarly higher than that of the general population in the United States. Third, we found that the risk of developing second primary solid cancer after radiotherapy increased with latency and tumor size. Fourth, in contrast to the trends for second primary solid tumors, the radiotherapy-correlated risk of developing second primary gastrointestinal cancers declined with age at diagnosis.

Previous research has demonstrated that radiotherapy enhanced the probability of particular SPMs in various primary malignancies. For instance, a study enrolling testicular cancer survivors from 1947 to 1991 found that patients with testicular cancer who underwent radiotherapy had a 2.9-fold increased risk of developing pancreatic cancer than those who did not receive radiotherapy, which the risk persisted for over twenty years [24]. Enrolling women with nonmetastatic breast from 1965 to 1989, Kaufman et al. demonstrated that the radiotherapy-correlated risk of developing second primary lung cancer increased in breast cancer patients, especially for ipsilateral lung among ever-smokers [25]. For patients with primary lung cancer, the annual rate of acquiring an SPLC was 1.10% per patient, with young women having an especially high-risk [19]. However, there has been conflicting evidence regarding how radiotherapy affects the emergence of SPMs in patients with primary lung cancer. Gonzalez et al. discovered that radiation enhanced NSCLC patients' likelihood of acquiring SPMs, which concentrated on all patients over 20 years old with lung cancer of all stages and was based on data from 1973 to 2002 [20]. According to a meta-analysis, Burdett et al. have shown that postoperative radiotherapy led to an 18% relative increase in the risk of death for NSCLC patients, indicating a negative effect caused by postoperative radiotherapy [21]. Our investigation, which was based on a sizable population with lung cancer, showed results that were consistent with the idea that radiotherapy was to blame for the occurrence of secondary solid tumors. And we found that the risk of second primary gastrointestinal malignancies was increasing after the implementation of radiotherapy. On the other hand, using the data from 1975 to 2011, Han et al. have revealed that radiotherapy was considerably related to a low risk of developing SPMs in lung cancer patients, especially second primary prostate cancer and thyroid cancer [22]. Han et al. also showed a significantly increased risk for second primary esophageal carcinoma in lung cancer patients, which was similar to our conclusions. The aforementioned studies of primary lung cancer were unable to thoroughly assess the risk of radiation for SPMs in comparison to research examining SPMs in individuals with other primary malignancies. Several factors, including differences in inclusion and exclusion criteria, number of patients, definition of SPMs, and methodology, were assumed to account for the inconsistency of the results in earlier publications.

Currently, the primary treatment for non-metastatic lung cancer is surgery-based combination therapy, followed by adjuvant chemotherapy or radiotherapy [26]. According to the latest ASCO guideline, adjuvant radiotherapy was considered for NSCLC patients with N2 disease but was not recommended for resected stage I or II NSCLC [27]. Our study focused on the effects of radiotherapy on surgically treated patients with non-metastatic lung cancer, aiming to evaluate potential risks of irradiation for developing SPMs. Compared to earlier research that had clear inclusion criteria, we have reached more reliable conclusions and offered stronger evidence in favor of updating guidelines. Because the incidence of developing SPMs was fairly low compared to other adverse events, a broader population was investigated in this investigation than in past studies to increase the trustworthiness of the results [20]. Additionally, radiotherapy was found to be insignificantly linked with second hematologic malignancies in lung cancer, despite being a risk factor for second hematologic malignancies in other cancers, such as prostate cancer [28]. The risk of SPMs was also evaluated using a variety of statistical techniques, such as Poisson regression and competing risk regression. Both approaches consistently showed that the probability of acquiring gastrointestinal cancer and second primary solid cancer was enhanced by radiotherapy. We also computed the SIRs for external validation and compared the incidence of SPMs among lung cancer survivors to the general population of the United States, which may broaden the interpretive perspective on the relationship between irradiation and the development of SPMs.

We divided patients into various subgroups based on their clinical characteristics to precisely assess the potential risk of radiotherapy, and we discovered an elevated risk of acquiring second primary solid and gastrointestinal malignancies in the majority of subgroups. And of all cancers, primary gastrointestinal cancer was the most common. In earlier research, the latency cut-off point varied and the prevalence of SPMs was associated with the latency [29]. We have used the minimal latency period for radiation-induced tumorigenesis in the analysis [23]. The highest radiotherapy-correlated risk was observed after a latency of more than ten years in both primary solid cancer and gastrointestinal cancer, suggesting that long-term follow-up may be essential for resectable lung cancer patients receiving radiotherapy. Next, we examined the radiotherapy-correlated risk in various genders and discovered that men had a higher relative risk of developing second primary solid malignancies following radiotherapy, but women had a higher relative risk of developing second primary esophageal cancer. The prevalence of esophageal cancer was much higher in males than in females, consistent with global statistics, but the proportion of second primary esophageal cancer was higher in females [3]. Moreover, our findings indicated that the risk of developing second primary gastrointestinal cancer decreased with increasing age at diagnosis, whereas the risk of developing second primary solid cancer was higher in patients younger than fifty and older than seventy. More specific screening for second primary solid cancer is advised for both young and elderly people, whereas screening for gastrointestinal cancer is recommended for those under 70 years old.

The merits of this study have been distinctly observed. Our study was based on a large population with relatively homogenous treatment exposure, which could make the results reliable and solid. Furthermore, we have evaluated the radiotherapy-correlated risk of developing individual SPMs for lung cancer, which was rarely studied and had significant implications in lung cancer treatment. Besides, different statistical methods were used in the study to comprehensively validate the results. Certainly, there are some limitations in this study. First, due to the incomplete records of the SEER database, it was unclear whether delayed radiotherapy could lead to the underestimation of the radiotherapy-correlated risk. Second, as a population-based study, potential biases may be caused by the lack of randomization of the first treatment. And the development of SPMs might be affected by other risk factors, including other treatments, environmental factors and genetic characteristics [30]. Third, the SEER database lacked detailed information on radiotherapy, such as the number of doses administered, which may limit the analysis of the specific relationship between radiotherapy and SPMs.

## Conclusions

To summarize, SPMs are uncommon but important adverse events after receiving radiotherapy, which is rarely investigated in lung cancer treatment. Based on a large population with resectable lung cancer, our study used different methodologies to comprehensively evaluate the relationship between radiotherapy and SPMs, assessing the elevated risk of developing second primary solid and gastrointestinal cancers. Our findings could serve as a meaningful reference for the early detection and treatment of SPMs in patients with resectable lung cancer receiving radiotherapy. Randomized controlled trials should be conducted in the future to further validate our conclusion, and further subgroup analysis is needed to evaluate the radiotherapy-correlated risk of different subpopulations.

## Supplementary Information

**Additional file 1.** Additional figures and tables.

## Data Availability

All the data are available in a public, open-access repository.

## Abbreviations

NSCLC: Non-small cell lung cancer
SPM: Second primary malignancy
SEER: Surveillance, epidemiology and end results
RR: Relative risk
SIR: Standardized incidence ratio
CI: Confidence interval
HR: Hazard ratio
