# Supplementary material for: Association between radiotherapy and risk of second primary malignancies in patients with resectable lung cancer: a population-based study
Source: J Transl Med. 2023 Jan 9;21:10. doi: 10.1186/s12967-022-03857-y (PMC9827664; doi:10.1186/s12967-022-03857-y)
Supplement: Supplementary file 1 — Additional file 1. Additional figures and tables. [file 12967_2022_3857_MOESM1_ESM.pdf]

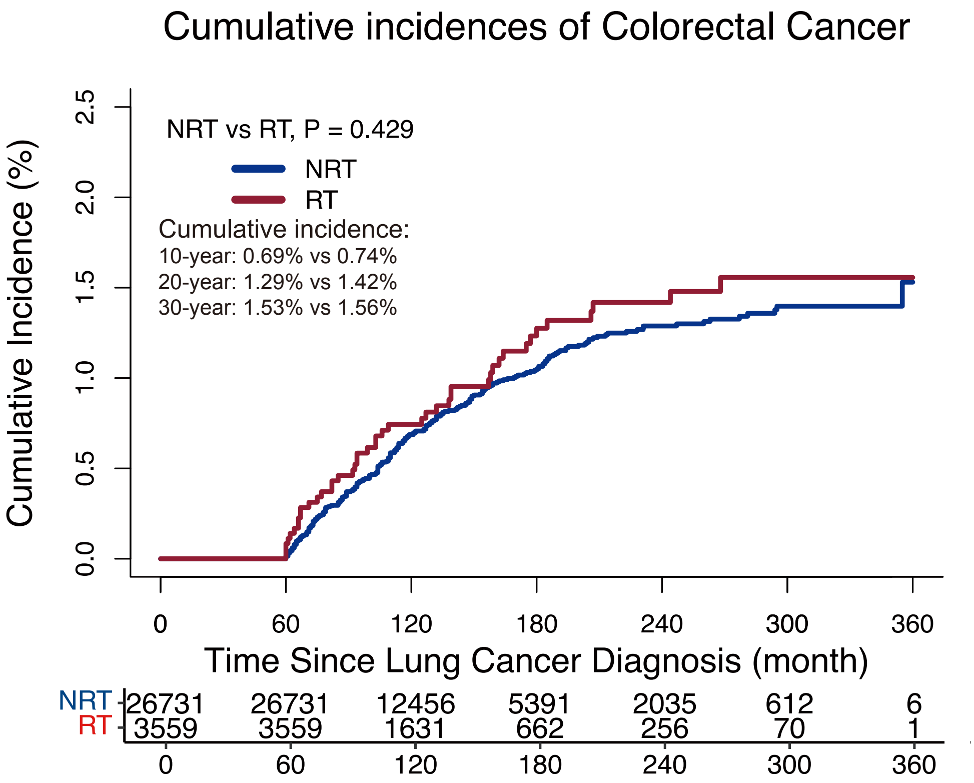

Figure S1. The comparisons of cumulative incidence of second primary colorectal cancer between subgroups stratified by radiotherapy use. The Fine-Gray test determined the P values. RT, radiotherapy; NRT, no radiotherapy.

Characteristics	RT/NRT cases	RR (95%CI)
Gender		
Male	20/42	2.85 (1.79–4.44)
Female	9/13	6.04 (2.87–12.33)
Race		
White	25/45	3.69 (2.41–5.57)
Black	2/6	2.35 (0.49–8.25)
Other	2/4	2.48 (0.49–9.99)
Age at diagnosis		
20–49	3/4	2.98 (0.78–10.58)
50–69	25/39	3.84 (2.49–5.84)
≥70	1/12	0.97 (0.10–3.98)
Histology		
Adenocarcinoma	12/23	4.90 (2.65–8.74)
Squamous cell carcinoma	13/26	2.25 (1.24–3.95)
Other	4/6	2.98 (0.94–8.74)
Tumor size		
≤2cm	5/17	4.53 (1.80–9.99)
>2cm	23/35	3.39 (2.14–5.28)
Unknown	1/3	0.82 (0.07–4.67)
Follow-up time (months)		
60 to 119	14/37	2.40 (1.39–3.98)
120 to 239	15/18	5.46 (3.01–9.81)

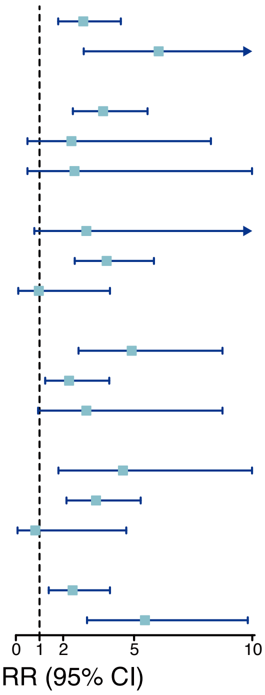

Figure S2. The relative risk and 95% confidence interval for second primary esophageal cancer among subgroups stratified by different characteristics. RR, relative risk; CI, confidence interval; RT, radiotherapy; NRT, no radiotherapy.

Characteristics	RT/NRT cases	RR (95%CI)
Gender		
Male	10/32	2.16 (1.14–3.85)
Female	4/17	2.55 (0.91–6.01)
Race		
White	7/44	1.32 (0.63–2.49)
Black	2/5	16.80 (4.55–81.61)
Other	2/3	4.24 (0.79–19.49)
Age at diagnosis		
20–49	1/1	4.39 (0.33–58.86)
50–69	11/26	2.68 (1.44–4.77)
≥70	2/22	1.17 (0.27–3.36)
Histology		
Adenocarcinoma	2/26	0.81 (0.19–2.31)
Squamous cell carcinoma	5/19	1.72 (0.68–3.78)
Other	4/7	9.06 (3.23–27.95)
Tumor size		
≤2cm	2/14	2.61 (0.58–7.86)
>2cm	10/33	1.94 (1.02–3.46)
Unknown	2/2	2.67 (0.47–15.32)
Follow-up time (months)		
60 to 119	8/31	2.09 (1.03–3.90)
120 to 239	5/17	2.23 (0.88–4.98)
240 to 360	1/1	7.55 (0.54–105.61)

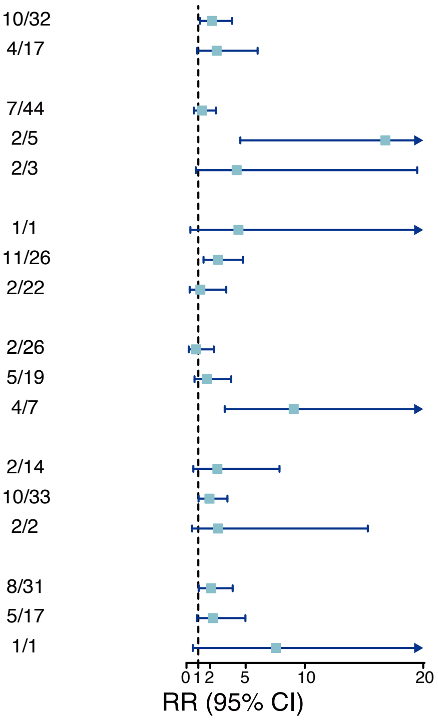

Figure S3. The relative risk and 95% confidence interval for second primary stomach cancer among subgroups stratified by different characteristics. RR, relative risk; CI, confidence interval; RT, radiotherapy; NRT, no radiotherapy.

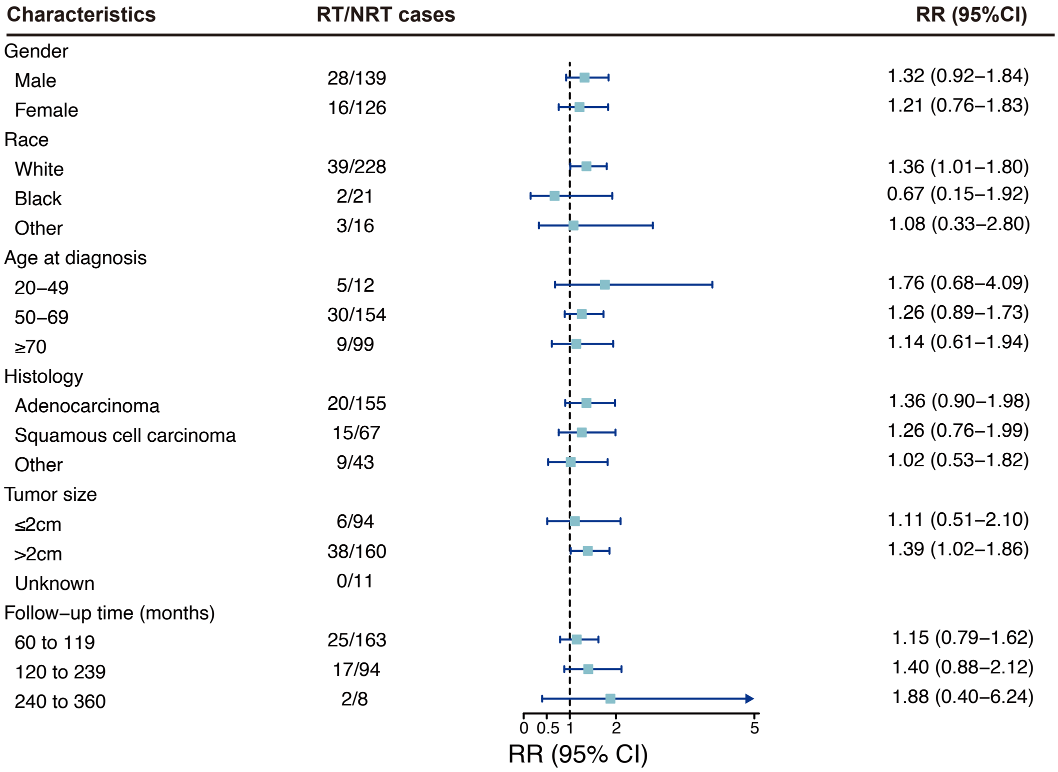

Figure S4. The relative risk and 95% confidence interval for second primary colorectal cancer among subgroups stratified by different characteristics. RR, relative risk; CI, confidence interval; RT, radiotherapy; NRT, no radiotherapy.

Table S1. The definition of different cancer used in this study

Site of Second Primary Malignancy	ICD-O-3 Site	Site recode ICD-O-3/WHO 2008	ICD-O-3 Histology (Type)
Main solid malignancies			
Breast cancer	C500-C509		All excluding 9050-9055, 9140, 9590-9992
Gastrointestinal cancer	C150-C159, C160-C169, C180-189, C260, C199, C209		All excluding 9050-9055, 9140, 9590-9992
Colon and Rectum	C180-189, C260, C199, C209		All excluding 9050-9055, 9140, 9590-9992
Esophagus	C150-C159		All excluding 9050-9055, 9140, 9590-9992
Lung and Bronchus	C340-C349		All excluding 9050-9055, 9140, 9590-9992
Stomach	C160-C169		All excluding 9050-9055, 9140, 9590-9992
Kidney	C649		All excluding 9050-9055, 9140, 9590-9992
Liver	C220		All excluding 9050-9055, 9140, 9590-9992
Melanoma	C440-C449		8720-8790
Pancreas	C250-C259		All excluding 9050-9055, 9140, 9590-9992

Prostate	C619		All excluding 9050-9055, 9140, 9590-9992
Urinary bladder	C670-C679		All excluding 9050-9055, 9140, 9590-9992
Main hematologic malignancies			
Non-Hodgkin lymphoma		Non-Hodgkin Lymphoma	9590-9597, 9670-9671, 9673, 9675, 9678-9680, 9684, 9687- 9691, 9695, 9698-9702, 9705, 9708-9709, 9712, 9714-9719, 9724-9729, 9735, 9737-9738, 9759, 9811-9818, 9823, 9827, 9837
Myeloma		Myeloma	9731-9732, 9734
Leukemia		Leukemia	9826, 9835-9836, 9811-9818, 9837, 9840, 9861, 9865-9867, 9869, 9871-9874, 9895-9898, 9910-9911, 9920, 9891, 9863, 9875-9876, 9945-9946, 9860, 9930, 9801, 9805-9809, 9931, 9827

Table S2. The baseline characteristics of bronchus and lung patients in this study.

Characteristics	NRT (N=51094)	RT (N=11341)
**Chemotherapy**		
No/Unknown	45009 (88.1%)	5783 (51.0%)
Yes	6085 (11.9%)	5558 (49.0%)
**Tumor size**		
=<2cm	17570 (34.4%)	1751 (15.4%)
>2cm	32012 (62.7%)	8680 (76.5%)
Unknown	1512 (3.0%)	910 (8.0%)
**Tumor histology**		
Adenocarcinoma	29006 (56.8%)	5094 (44.9%)
Other	8521 (16.7%)	2631 (23.2%)
Squamous cell carcinoma	13567 (26.6%)	3616 (31.9%)
**Tumor histological stage**		
Localized	32341 (63.3%)	1575 (13.9%)
Regional	18753 (36.7%)	9766 (86.1%)
**Tumor grade**		
Grade I/II	24831 (48.6%)	3608 (31.8%)
Grade III/IV	18962 (37.1%)	6112 (53.9%)
Unknown	7301 (14.3%)	1621 (14.3%)
**Race**		
Black	4277 (8.4%)	1110 (9.8%)
Other	3404 (6.7%)	810 (7.1%)
White	43413 (85.0%)	9421 (83.1%)
**Gender**		
Female	24766 (48.5%)	4711 (41.5%)
Male	26328 (51.5%)	6630 (58.5%)
**Year of diagnosis**		
1975-1994	12137 (23.8%)	4102 (36.2%)
1995-2004	17985 (35.2%)	4380 (38.6%)
≥2005	20972 (41.0%)	2859 (25.2%)
**Age group**		
20-49	3347 (6.6%)	1176 (10.4%)
50-69	26732 (52.3%)	6970 (61.5%)
≥70	21015 (41.1%)	3195 (28.2%)
**Survival months**		
Median [Min, Max]	64.0 [0, 371]	30.0 [0, 371]

RT, radiotherapy; NRT, no radiotherapy.

Table S3. The baseline characteristics of bronchus and lung patients who developed second primary malignancies in this study.

Characteristics	Patients with 5-year survival		Patients with 2-year survival	
	NRT (N=1551)	RT (N=264)	NRT (N=314)	RT (N=56)
**Chemotherapy**				
No/Unknown	1420 (91.6%)	151 (57.2%)	289 (92.0%)	25 (44.6%)
Yes	131 (8.4%)	113 (42.8%)	25 (8.0%)	31 (55.4%)
**Tumor size**				
=<2cm	613 (39.5%)	33 (12.5%)	129 (41.1%)	12 (21.4%)
>2cm	891 (57.4%)	214 (81.1%)	173 (55.1%)	43 (76.8%)
Unknown	47 (3.0%)	17 (6.4%)	12 (3.8%)	1 (1.8%)
**Tumor histology**				
Adenocarcinoma	936 (60.3%)	120 (45.5%)	182 (58.0%)	23 (41.1%)
Other	220 (14.2%)	66 (25.0%)	43 (13.7%)	11 (19.6%)
Squamous cell carcinoma	395 (25.5%)	78 (29.5%)	89 (28.3%)	22 (39.3%)
**Tumor histological stage**				
Localized	1152 (74.3%)	36 (13.6%)	229 (72.9%)	8 (14.3%)
Regional	399 (25.7%)	228 (86.4%)	85 (27.1%)	48 (85.7%)
**Tumor grade**				
Grade I/II	725 (46.7%)	72 (27.3%)	152 (48.4%)	20 (35.7%)
Grade III/IV	519 (33.5%)	153 (58.0%)	117 (37.3%)	31 (55.4%)
Unknown	307 (19.8%)	39 (14.8%)	45 (14.3%)	5 (8.9%)
**Race**				
Black	128 (8.3%)	26 (9.8%)	22 (7.0%)	3 (5.4%)
Other	92 (5.9%)	21 (8.0%)	14 (4.5%)	2 (3.6%)
White	1331 (85.8%)	217 (82.2%)	278 (88.5%)	51 (91.1%)
**Gender**				
Female	652 (42.0%)	79 (29.9%)	142 (45.2%)	21 (37.5%)
Male	899 (58.0%)	185 (70.1%)	172 (54.8%)	35 (62.5%)
**Year at diagnosis**				
1975-1994	555 (35.8%)	106 (40.2%)	86 (27.4%)	20 (35.7%)
1995-2004	672 (43.3%)	125 (47.3%)	132 (42.0%)	22 (39.3%)
≥2005	324 (20.9%)	33 (12.5%)	96 (30.6%)	14 (25.0%)
**Age group**				
20-49	103 (6.6%)	33 (12.5%)	13 (4.1%)	3 (5.4%)
50-69	1000 (64.5%)	182 (68.9%)	184 (58.6%)	47 (83.9%)
≥70	448 (28.9%)	49 (18.6%)	117 (37.3%)	6 (10.7%)
**Survival months**				
Median [Min, Max]	158 [60.0, 360]	152 [60.0, 342]	118 [26.0, 336]	118 [26.0, 312]

RT, radiotherapy; NRT, no radiotherapy.

Table S4. The risk of developing second primary malignancies in patients with bronchus and lung cancer by multivariable competing risk regression.

Site of Second Primary Malignancy	HR	95%CI low	95%CI high	P value
Main solid malignancies	1.16	1.04	1.29	0.03
Breast cancer	0.92	0.68	1.23	0.62
Gastrointestinal Cancer	1.69	1.39	2.06	<0.01
Colon and Rectum	1.22	0.93	1.59	0.24
Esophagus	3.31	2.25	4.87	<0.01
Stomach	2.18	1.32	3.59	0.01
Kidney	1.30	0.76	2.23	0.42
Liver	0.35	0.13	0.94	0.08
Melanoma	1.11	0.66	1.87	0.73
Pancreas	1.29	0.81	2.05	0.36
Prostate	1.22	0.97	1.53	0.15
Urinary bladder	0.99	0.73	1.33	0.93
Main hematologic malignancies	1.04	0.82	1.32	0.78
Non-Hodgkin lymphoma	1.15	0.85	1.55	0.45
Myeloma	0.52	0.24	1.12	0.16
Leukemia	1.17	0.74	1.86	0.57

HR was adjusted by age and gender. HR, hazard ratio; CI, confidence interval.

Table S5. Standardized incidence ratio (SIR) for all second primary malignancies in RT and NRT subgroups of bronchus and lung cancer.

Site of Second Primary Malignancy	Patients received radiotherapy			Patients didn't receive radiotherapy		
	SIR	95%CI low	95%CI high	SIR	95%CI low	95%CI high
5-year latency						
Main solid malignancies	1.30#	1.15	1.47	1.08#	1.03	1.14
Breast cancer	0.98	0.68	1.37	0.97	0.86	1.08
Gastrointestinal Cancer	2.08#	1.67	2.55	1.16#	1.05	1.28
Colon and Rectum	1.35	0.99	1.81	1.07	0.95	1.20
Esophagus	8.14#	5.57	11.49	2.09#	1.59	2.69
Stomach	2.12#	1.09	3.70	1.13	0.84	1.48
Kidney	1.19	0.59	2.13	0.98	0.76	1.25
Liver	0.67	0.14	1.96	1.47#	1.08	1.97
Melanoma	0.84	0.42	1.50	0.85	0.68	1.05
Pancreas	1.98#	1.19	3.09	1.11	0.90	1.37
Prostate[a]	0.92	0.71	1.18	0.89	0.80	0.99
Urinary bladder	1.68#	1.15	2.37	1.77#	1.57	2.00
2-year latency						
Main hematologic malignancies	1.41#	1.10	1.79	1.06	0.96	1.16
Non-Hodgkin lymphoma	1.58#	1.12	2.17	1.12	0.98	1.28
Myeloma	0.68	0.25	1.47	0.87	0.66	1.12
Leukemia	1.57#	1.02	2.30	1.06	0.90	1.25

a, only men were included for analysis. RT, radiotherapy; NRT, no radiotherapy. #P value < 0.05.
